# Body Composition in Severe Refractory Asthma: Comparison with COPD Patients and Healthy Smokers

**DOI:** 10.1371/journal.pone.0013233

**Published:** 2010-10-06

**Authors:** Markos Minas, Andriana I. Papaioannou, Agori Tsaroucha, Zoe Daniil, Chrissi Hatzoglou, Markos Sgantzos, Konstantinos I. Gourgoulianis, Konstantinos Kostikas

**Affiliations:** 1 Respiratory Medicine Department, University of Thessaly Medical School, Larissa, Greece; 2 Department of Physiology, University of Thessaly Medical School, Larissa, Greece; 3 Department of Anatomy, University of Thessaly Medical School, Larissa, Greece; Erasmus University Rotterdam, Netherlands

## Abstract

**Background:**

Body composition is an important parameter for patients with chronic obstructive pulmonary disease (COPD) whereas the association between asthma and obesity is not fully understood. The impact of severe refractory asthma (SRA) on fat free mass (FFM) has not been investigated.

**Methodology and Principal Findings:**

213 subjects (70 healthy smokers, 71 COPD patients and 72 asthma patients) without significant comorbidities were included in the study. In all patients, body composition assessment (using bioelectrical impendance analysis, skinfold and anthropometric measurements) and spirometry were performed. Differences in fat free mass index (FFMI) between groups were assessed and determinants of FFMI in asthma were evaluated. Patients with SRA had lower values of FFMI compared to patients with mild-to-moderate asthma [18.0(17.3–18.3)–19.5(18.4–21.5), p<0.001], despite the fact that they were more obese. The levels of FFMI in SRA were lower than those of GOLD stage I–III COPD and comparable to those of stage IV COPD patients [18.0(17.3–18.3)–18.8(17.8–20.1), p = ns]. These differences were present even after proper adjustments for sex, age, smoking status, daily dose of inhaled corticosteroids (ICS) and daily use of oral corticosteroids (OCS). In multivariate analysis, independent predictors of FFMI in asthmatic patients were age, use of OCS and the presence of SRA, but not smoking, sex or cumulative dose of ICS used.

**Conclusions and Significance:**

SRA is related to the presence of low FFMI that is comparable to that of GOLD stage IV COPD. The impact of this observation on asthma mechanisms and outcomes should be further investigated in large prospective studies.

## Introduction

Body composition is an important parameter for humans as it has been shown that either malnutrition or obesity, both expressed as body mass index (BMI), are correlated with overall morbidity and mortality [1]. Previous studies from the general population indicate that high values of body fat (BF) and low values of fat free mass (FFM) are independent predictors of all cause mortality [2]. Moreover, it was shown that in healthy subjects total BF, central adiposity and FFM are associated with lung function [3], whereas central adiposity seems to be a better predictor of lung function compared to body weight or BMI [4]. Finally, an 8-year study in the general population has shown that loss of weight and decrease in BMI is associated with an increase in lung function [5].

Body composition represents a risk factor for the development of both chronic obstructive pulmonary disease (COPD) and asthma [6], [7]. Men with low values of BMI are at increased risk for the development of COPD [8]. Moreover, although it is unclear if malnutrition is an independent risk factor for COPD development, malnutrition and weight loss can reduce respiratory muscle mass and the strength of the remaining muscle fibers [6]. On the contrary, obesity represents a risk factor for asthma development [7]. It has been shown that increase in BMI values predisposes to a new asthma diagnosis in female young adults [9], and high values of BMI increase the odds of incident asthma in both men and women [10].

On the other hand, smoking, COPD and asthma affect body composition. There is evidence that smoking induces loss of body weight, whereas smoking cessation provokes weight gain. Moreover, smoking contributes in the accumulation of visceral fat and in the development of insulin resistance, metabolic syndrome and type 2 diabetes [11]. Additionally, BMI and fat free mass index (FFMI) are predictors of mortality in patients with COPD [12] and lower values of BMI and FFMI are associated with increased risk of death in patients with COPD [13], [14]. In patients with asthma, weight and BMI are associated with asthma control, and overweight asthma patients are more difficult to achieve control [15]. Several hypotheses have been raised for the understanding of the association between asthma and obesity, including mechanical impairment, the presence of comorbidities and systemic inflammation and the role of adipose tissue hormones [16]. In contrast to COPD patients, body composition, and especially FFM, has not been extensively evaluated in patients with asthma.

The aim of the present study was to assess body composition in patients with asthma and compare it to COPD patients and healthy smokers. We tested the hypothesis that patients with severe refractory asthma (SRA) might have lower FFMI than those with mild-to-moderate asthma. Predictors of FFMI in patients with asthma were further evaluated.

## Materials and Methods

### Study design

Study participants were asthmatic patients, COPD patients and healthy smokers, without significant comorbidities that might affect body composition, including history of cardiovascular disease (CVD), diabetes mellitus, history of cancer, other lung disease or any other chronic systemic disease. COPD and asthma patients were recruited from the outpatients clinics of a tertiary hospital and had stable disease for the previous 8 weeks. Diagnosis of COPD and asthma has set at least one year before the inclusion in the study by an expert respiratory physician (K.K.) with specific interest in COPD and asthma, according to the existing guidelines [6], [7]. Healthy smokers were divided to current and ex smokers (stopped smoking for at least one year prior to their inclusion).

A structured questionnaire was completed for all study participants, including demographic data, as well as data regarding their disease (for asthmatic and COPD patients) e.g. duration of treatment, use of inhaled corticosteroids (ICS), total ICS dose per day expressed in equivalent mcg of beclomethasone, number of exacerbations during the past year which were treated with short courses of oral corticosteroids (OCS), and daily dose of OCS for some patients with SRA.

All subjects additionally completed a food frequency questionnaire (FFQ), which has been used in the Greek population, for the assessment of their nutritional status [17], [18]. All subjects were submitted to body composition assessment, skinfolds measurements, assessment of anthropometric parameters and simple spirometry. The study protocol was approved by the review board of the University Hospital of Larissa and all patients provided written informed consent.

### Body composition, skinfold and anthropometric measurements

Body composition was assessed by bioelectrical impendance analysis (BIA), with a commercially available body analyzer (BIA 101 System Analyser, Akern, Florence, Italy) according to current recommendations [19], [20]. FFMI was calculated as FFM/height squared. Skinfolds were measured using a commercially available calliper (GIMA, Plicometro skinfold calliper mechanical, San Donato Milanese, Italy). Skinfolds measured were: biceps, triceps, chest, subscapular, midaxillary, abdominal, suprailiac, thigh and calf skinfold. Anthropometric circumferences and diameters were measured using a commercially available measuring tape. Measurements included the following parameters: neck circumference, chest circumference, waist circumference, abdominal circumference at the umbilicus level, circumference anteriorly between the xiphoid process of the sternum and the umbilicus and laterally between the lower end of the rib cage and iliac crests, iliac circumference, iliac diameter, hip circumference and knee circumference. All body composition measurements were performed by the same experienced technician who was blinded to the status of the study participants

### Spirometry

All patients were submitted to spirometry by the same experienced technician, using a commercially available system (Master Screen, Erich Jaeger GmbH, Wuerzburg, Germany) according to American thoracic society (ATS) guidelines [21]. Post-bronchodilator values were recorded for patients with asthma and COPD.

### Classification of Patients with Asthma and COPD

Patients with asthma were classified as mild-to-moderate asthma patients and patients with SRA according to the ATS and global initiative for asthma (GINA) guidelines [7], [22], [23]. COPD patients were distributed to the stages of the disease according to their forced expiratory volume in the 1^st^ second (FEV_1_), as it is referred in the global initiative for chronic obstructive pulmonary disease (GOLD) guidelines (Stage I – mild COPD FEV_1_≥80.0% predicted; Stage II – moderate COPD 50.0%≤FEV_1_<80.0% predicted; Stage III – severe COPD 30.0%≤FEV_1_<50.0%; Stage IV – very severe COPD FEV_1_≤30.0% or FEV_1_<50.0% predicted with respiratory failure) [6].

### Statistical analysis

Normally distributed data are presented as mean±SD whereas skewed data are presented as median (interquartile range). Normality of distribution of the variables was assessed using the D'Agostino-Pearson normality test. Comparisons of variables between two groups was performed using unpaired Student's t-tests or Mann-Whitney U tests for normally distributed and skewed variables, respectively whereas comparisons of variables between three or more groups was performed using one way ANOVA or Kruskal Wallis test for normally distributed and skewed variables, respectively, with suitable post hoc analyses (Bonferroni or Dunn's, respectively) for all pairs of data. For the comparison of FFMI among all groups of subjects a univariate general linear model with appropriate adjustment for confounding factors (sex, age, smoking habit, ICS dose and daily use of OCS) was used; the equality of variances was assessed with Levere's test and post hoc analysis was performed with Dunnett's T3 test. Pearson and Spearman's rank correlation coefficients were used for the correlation between normally distributed and skewed variables, respectively. Multiple regression analysis was performed for the evaluation of FFMI predictors, using FFMI as dependent variable and sex, age, smoking habit, cumulative ICS dose, daily use of OCS and asthma classification (mild-to-moderate vs. SRA) as independent variables. Stepwise multiple regression analysis was additionally used for the development of a prediction equation for FFM in patients with asthma, using anthropometric parameters. P values<0.05 were considered statistically significant. Analyses were performed using GraphPad Prism 5 (GraphPad Software Inc, La Jolla, CA, USA) and SPSS 16 (SPSS, Chicago, IL, USA).

## Results

In total, 213 patients were included in the study. Of these 70 were healthy smokers, 71 were COPD patients and 72 were asthma patients. Demographic data for each group are presented in Table 1. Smokers and COPD patients were mainly males, whereas the asthma group was composed mainly by females. Patients with asthma had higher values of BMI, fat mass (FM) and fat mass index (FMI) (Table 1). All asthma patients were receiving ICS compared to the 47.9% of COPD patients. Moreover, 9 (12.5%) patients with asthma were receiving OCS daily, all with SRA. Table 2 shows the nutritional status of each group as it was assessed with a FFQ and suggests that subjects in the three groups had similar nutritional status and were not malnourished.

**Table 1:** Demographic data of the three groups of study participants.

	Smokers	COPD	Asthma
	(n = 70)	(n = 71)	(n = 72)
Male/Female	58/12	49/22	18/54
Age (years)	56 (49–69)	70 (65–77)	61.5 (51–73.5)
Smoking status			
Current smokers	48 (68.6%)	26 (36.6%)	3 (4.2%)
Ex smokers	22 (31.4%)	45 (63.4%)	3 (4.2%)
Non smokers	0	0	66 (91.6%)
PYS	40 (22–60)	65 (50–90)	30 (20–40)
ICS	-	34 (47.9%)	72 (100%)
ICS dose (mcg)	-	1000 (1000–2000)	1000 (1000–2000)
Daily OCS (n)	-	-	9
Courses of OCS during the past year	-	19 (26.8%)	17 (23.6%)
FEV_1_ (%)	97.7±11.8	57.1±8.6	88.0±18.7
FVC (%)	98.1±12.5	76.0±19.0	93.6±16.7
FEV_1_/FVC (%)	79.4±4.9	57.7±11.3	74.3±11.8
PEF (%)	89.6±19.6	57.7±20.6	75.6±20.2
FEF_25–75_ (%)	85.6±25.7	28.1±14.9	59.4±32.6
BW (kg)	85.0 (75.0–92.0)	79.0 (70.0–90.0)	86.5 (75.0–98.5)
BMI (kg/m^2^)	28.5 (26.5–31.1)	27.0 (25.2–30.9)	33.2 (29.1–36.3)
FM (kg)	19.2 (16.8–22.3)	18.5 (12.7–23.8)	36.2 (23.3–47.9)
FMI (kg/m^2^)	6.7 (5.4–7.8)	6.5 (4.6–8.2)	14.8 (8.8–17.8)
FFM (kg)	65.3 (61.1–72.0)	58.1 (54.1–67.4)	48.5 (44.3–57.8)
FFMI (kg/m^2^)	22.1 (20.3–23.5)	20.7 (19.0–23.7)	18.8 (17.9–20.7)

Values are reported as mean ± SD or median (interquartile ranges) for normally distributed and skewed data, respectively.

PYS: Pack Years, ICS: Inhaled corticosteroids, OCS: Oral corticosteroids, BW: Body Weight, BMI: Body Mass Index, FM: Fat Mass, FMI: Fat Mass Index, FFM: Fat Free Mass, FFMI: Fat Free Mass Index, FEV_1_: Forced Expiratory Volume in the 1^st^ second, FVC: Forced Vital Capacity, PEF: Peak Expiratory Flow, FEF_25–75_: Forced mid-Expiratory Flow.

**Table 2:** Food items consumed (in servings/week) in each group.

	Smokers	COPD	Asthma	p-value
Red meat	1 (1–2)	1 (1–2)	1 (0–2)	0.856
Pork	1 (1–2)	1 (0–2)	1 (0–2)	0.394
Poultry	1 (1–2)	1 (1–2)	1 (0–2)	0.056
Fish	1 (1–2)	1 (1–2)	1 (1–2)	0.503
Egg	1 (1–3)	1 (0–2)	1 (0–2)	0.128
Bread and cereals	7 (3–7)	7 (5–7)	7 (6–7)	0.108
Pasta and rice	2 (1–3)	2 (1–3)	3 (1–4)	0.677
Potatoes	2 (2–3)	2 (1–3)	3 (1–3)	0.338
Vegetables	7 (4.5–7)	7 (4.5–7)	7 (5–7)	0.575
Fruit and juices	6 (4–7)	5 (3–7)	5 (4–7)	0.999
Milk and products full fat	2 (0–4.5)	5 (0–7)	5 (0–7)	0.213
Milk and products low fat	0 (0–0)	0 (0–0)	0 (0–0)	0.474
Cheese yellow	1 (0–4)	0 (0–2)	0 (0–3)	0.079
Cheese white	6 (4–7)	4.5 (3–7)	5 (4–7)	0.150
Legumes	5 (3–5.5)	4 (2–5)	5 (3–6)	0.269
Desert or ice cream	1 (0–3)	1 (0–2)	2 (1–3)	0.157

Values are reported as median (interquartile ranges).

### Body Composition in Healthy Smokers and Patients with COPD and Asthma

Regarding the group of healthy smokers, ex smokers had higher values of body weight, BMI and FFMI compared to current smokers (Table 3). Patients with stage IV COPD had lower values of FFM and FFMI compared to the earlier stages of the disease, whereas the values of FM and FMI were similar in all stages of the disease (Table 4). Regarding asthma patients, patients with SRA had higher values of BMI, FM and FMI and lower values of FFMI (Table 5).

**Table 3:** Body composition in ex and current healthy smokers.

	Ex smokers(n = 22)	Current smokers(n = 48)	p-value
BW (kg)	90.0 (80.0–92.0)	83.0 (71.3–89.3)	0.038
BMI (kg/m^2^)	31.1 (27.5–33.8)	27.3 (26.1–29.8)	0.003
FM (kg)	20.4 (17.2–23.2)	19.1 (15.8–21.7)	0.235
FMI (kg/m^2^)	6.9 (5.8–8.0)	6.5 (5.0–7.6)	0.194
FFM (kg)	68.0 (62.7–75.8)	65.0 (59.4–71.8)	0.143
FFMI (kg/m^2^)	23.5 (21.7–26.9)	21.7 (20.0–23.0)	0.008

Values are reported as median (interquartile ranges).

BW: Body Weight, BMI: Body Mass Index, FM: Fat Mass, FMI: Fat Mass Index, FFM: Fat Free Mass, FFMI: Fat Free Mass Index.

**Table 4:** Body composition in COPD patients according to GOLD stages of the disease.

	Stage I	Stage II	Stage III	Stage IV	p-value
	(n = 9)	(n = 32)	(n = 19)	(n = 11)	
BW (kg)	92.0 (78.0–105.0)	79.0 (70.0–89.3)	75.0 (66.0–80.0)	76.0 (67.0–83.0)	0.073
BMI (kg/m^2^)	30.9 (26.8–34.3)	27.0 (25.2–30.3)	26.0 (24.8–29.4)	26.6 (23.4–28.9)	0.179
FM (kg)	18.1 (12.4–22.8)	18.3 (13.2–22.8)	16.1 (11.2–23.3)	24.0 (12.5–26.9)	0.619
FMI (kg/m^2^)	6.2 (4.4–7.3)	6.4 (4.8–8.1)	5.7 (4.1–9.2)	8.2 (4.4–9.4)	0.586
FFM (kg)	74.6 (63.8–79.7)	58.5 (54.6–67.8)∧	57.1 (53.6–62.9)∧	55.1 (48.8–57.7)∧	0.002
FFMI (kg/m^2^)	24.4 (22.1–26.9)	20.8 (19.4–23.8)	20.6 (19.3–22.8)	18.8 (17.8–20.1)∧	0.004

Values are reported as median (interquartile ranges).

BW: Body Weight, BMI: Body Mass Index, FM: Fat Mass, FMI: Fat Mass Index, FFM: Fat Free Mass, FFMI: Fat Free Mass Index.

∧ Indicate statistically significant difference with Stage I.

**Table 5:** Body composition in asthma patients according to their stage of the disease.

	Mild to moderate	Severe refractory asthma	p-value
	(n = 48)	(n = 24)	
BW (kg)	83.0 (69.3–89.5)	97.0 (87.0–103.8)	<0.001
BMI (kg/m^2^)	31.2 (27.3–34.0)	35.0 (34.6–37.4)	<0.001
FM (kg)	26.1 (22.3–37.4)	48.3 (41.7–54.3)	<0.001
FMI (kg/m^2^)	9.7 (7.6–14.9)	17.3 (16.8–19.4)	<0.001
FFM (kg)	49.9 (43.1–65.6)	48.2 (44.7–49.5)	0.199
FFMI (kg/m^2^)	19.5 (18.4–21.5)	18.0 (17.3–18.3)	<0.001

Values are reported as median (interquartile ranges).

BW: Body Weight, BMI: Body Mass Index, FM: Fat Mass, FMI: Fat Mass Index, FFM: Fat Free Mass, FFMI: Fat Free Mass Index.

**Figure 1:**
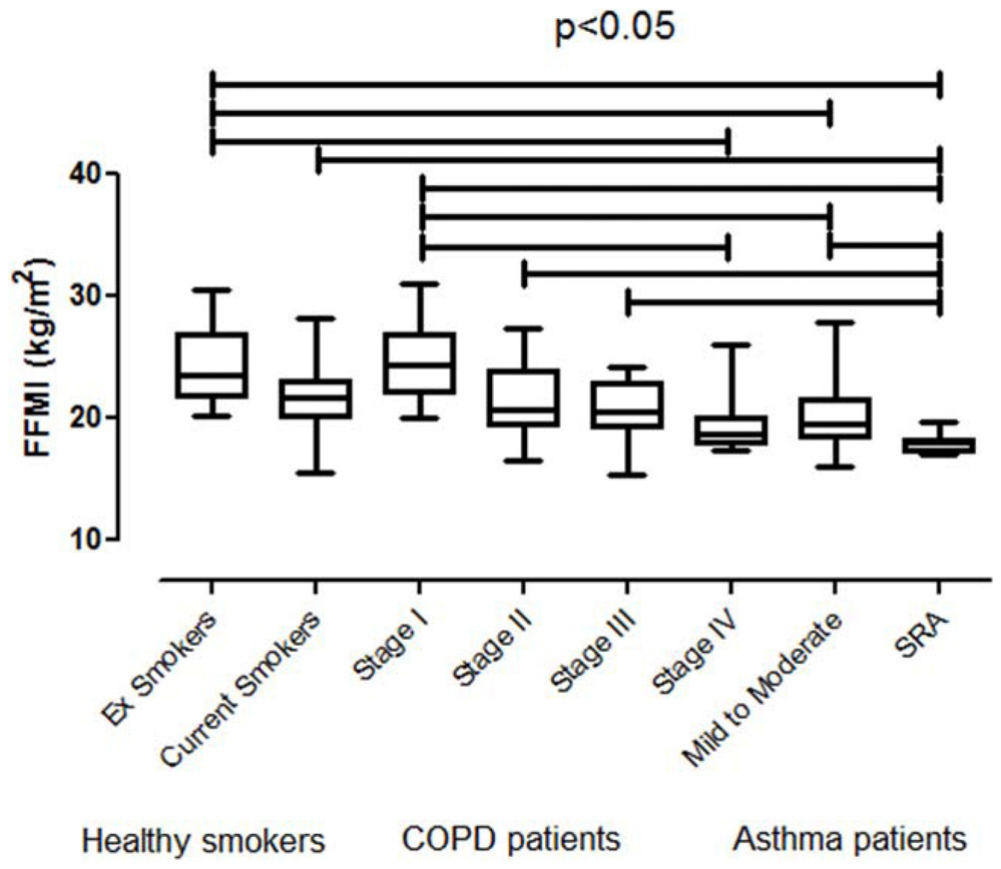
Comparison of FFMI between all subgroups of subjects. ES: Ex smokers, CS: Current smokers, MtM: Mild-to-moderate asthma, SRA: Severe refractory asthma.

Differences in FFMI in all subgroups of healthy smokers, COPD and asthma patients are presented in Figure 1. Patients with SRA had significantly lower FFMI compared to healthy current and ex smokers, as well as to patients with COPD stage I, II, III and patients with mild-to-moderate asthma, whereas their levels were comparable to those of stage IV COPD.

After proper adjustments for sex, age, smoking habit, ICS dose and daily use of OCS, patients with SRA still presented significantly lower FFMI compared to healthy current (p<0.001) and ex smokers (p<0.001), as well as to patients with COPD stage I (p = 0.007), II (p<0.001), III (p = 0.005), and patients with mild-to-moderate asthma (p = 0.002). Again, there was no difference between patients with SRA and COPD stage IV (p = 0.826). Moreover, after the adjustments, ex smokers differed significantly with COPD stage III (p = 0.02), whereas there was no statistically significant difference between COPD stage I and patients with mild to moderate asthma (p = 0.087). All the other differences presented in Figure 1 remained statistically significant.

### Predictors of FFMI in Patients with Asthma

Predictors of FFMI in patients with asthma, provided by multiple regression analysis, are presented in Table 6. Age, daily use of OCS and the presence of SRA, but not sex, smoking habit or the cumulative dose of ICS, were independent predictors of FFMI (R^2^: 0.960, Adjusted R^2^: 0.956).

**Table 6:** Parameters associated with FFMI in asthma patients.

	B*	SE	Beta#	95% CI for B	p-value
Sex	1.219	1.606	0.030	−1.997, 4.425	0.451
Age	0.223	0.026	0.696	0.171, 0.275	<0.001
Smoking	1.996	1.856	0.038	−1.721, 5.714	0.287
ICS dose	0.000	0.001	−0.034	−0.003, 0.002	0.698
Daily OCS	−4.548	1.691	−0.088	−7.935, 1.162	0.009
Stage^a^	4.566	1.304	0.341	1.955, 7.177	0.001

*Unstandardized coefficients, SE: Standard error,

# Standardized coefficients.

ICS: Inhaled corticosteroids, OCS: Oral corticosteroids, Stage^a^: mild to moderate asthma or SRA.

FFMI in patients with asthma was correlated with subscapular skinfold (r = 0.354, p = 0.004), midaxillary skinfold (r = 0.335, p = 0.007), neck circumference (r = 0.268, p = 0.039), waist circumference (r = 0.417, p = 0.001), iliac diameter (r = 0.411, p = 0.002) and knee circumference (r = 0.314, p = 0.025). Stepwise multiple regression analysis using anthropometric parameters as independent variables showed that FFM values can be predicted based on weight, height, abdominal and calf skinfold and iliac diameter (Unadjusted R^2^: 0.999, Adjusted R^2^: 0.998; Table 7).

**Table 7:** Prediction equations for FFM in asthma patients.

	B*	SE	Beta#	95% CI for B	p-value
Weight (kg)	0.275	0.045	0.519	0.171, 0.379	<0.001
Height (cm)	0.176	0.026	0.575	0.116, 0.236	<0.001
Skinfolds (cm)					
Abdominal	0.327	0.030	0.278	0.258, 0.397	<0.001
Calf	−0.265	0.097	−0.102	−0.489, −0.042	0.026
Anthropometry (cm)					
Iliac diameter	−0.266	0..075	−0.258	−0.440, −0.093	0.008

*Unstandardized coefficients, SE: Standard error,

# Standardized coefficients.

## Discussion

The results of the current study indicate that there are differences in body composition among specific groups of healthy smokers, COPD and asthma patients without comorbidities. Patients with SRA have lower values of FFMI compared to patients with mild-to-moderate asthma, despite the presence of higher values of BMI and FMI. Additionally, the levels of FFMI in SRA were lower than those of current and ex smokers and comparable to those with stage IV COPD. Finally, age, daily use of OCS and the presence of SRA were the only independent predictors of FFMI in patients with asthma.

This is the first study to show that patients with SRA, despite the fact that they are often obese, have also reduced values of FFMI. Moreover, it was shown that SRA is an independent predictor of FFMI in asthma patients. These patients had similar values of FFMI compared to patients with stage IV COPD, although they had higher values of BMI. Shore has recently proposed several mechanisms which may interfere in the association between obesity and asthma, such us mechanical factors, systemic inflammation and the role of comorbidities [16]. On the other hand it is known that patients with SRA present chronic airway inflammation, with a possible activation of neutrophils [22]. It is additionally known that obesity provokes systemic inflammation, leading to the development of metabolic syndrome, insulin resistance and high levels of CRP [24]. The coexistence of intense local and systemic inflammation in these patients may lead to loss of FFM in patients with SRA.

The fact that the presence of SRA was an independent predictor of FFMI in asthmatics further supports the significance of body composition assessment in that population. The results of our study indicate that in patients with SRA, FFMI was also associated with the use of OCS, whereas there was no association with daily ICS dose. In a similar study by Targowski et al., it was shown that systemic corticosteroids lead to loss of FFM in the lower limbs, whereas ICS do not affect FFM [25]. Our data further support the role of OCS, but not of ICS, on body composition of asthmatic patients, besides the role of age and the presence of severe disease.

On the other hand, body composition has been widely studied in COPD and the extrapulmonary effects of this disorder that leads to cachexia and loss of FFM have been well established [6]. Our data replicate the findings of Ischaki et al. showing lower FFMI in COPD patients with stage IV COPD compared to those with stages 0 and I of the disease [26], since in the present study patients at stage III and IV had lower values of FFMI compared to patients at stage I of the disease. Regarding the loss of FFM in COPD patients, several hypotheses have been suggested which imply that COPD affects both structure and function of skeletal muscles [27]. These effects of the disease in skeletal muscle mass are very important, as it has been shown that FFM is an independent predictor of mortality, irrespective of FM [28]. However, there are data implying that the loss of FFMI may be attributed in dietary problems in COPD patients [29]. The results of the current study, however, indicate that patients with severe and very severe COPD have lower values of FFMI compared to patients with milder disease, despite the fact that our COPD patients had similar nutritional status compared to healthy smokers and asthma patients.

We have additionally shown that anthropometric and skinfolds methods may be used for the estimation of FFM in patients with asthma. Despite the fact that there were no strong correlations between single skinfolds and/or circumferences, we have shown that a combination of them may be used for the estimation of FFM in these patients. Equations using combinations of skinfold and/or anthropometric parameters have been used for body composition assessment in specific populations [30], [31]. In a previous study, Peterson et al. have developed equations using skinfold measurements for the prediction of per cent BF (%BF) with a 4 compartment model [32]. In healthy subjects weight to height ratio was the best predictor of BF [33], whereas in COPD patients, anthropometry has been studied for the estimation of body composition [34], [35]. To the best of our knowledge, this is the first study to assess body composition using skinfolds and anthropometric parameters in patients with asthma, and the equation provided from this study needs to be further validated in an independent population.

However, although the strength of the study is the careful selection of patients without comorbidities, this study has limitations. A significant limitation is that there were differences between the three groups concerning age, smoking status and gender distribution. In the healthy smokers and COPD groups subjects were predominantly male, whereas the asthmatics were predominantly female. Moreover COPD patients were older compared to asthma patients and healthy smokers. However, these differences are attributed to the prevalence of the diseases in the two genders, since it is known that SRA is more prevalent in women whereas, in Greece, smokers and COPD patients are mainly male [22], [36]. Another possible limitation of our study is the high prevalence of obesity and overweight in our population. However, the prevalence of obesity in the Greek population is one of the highest among Europe countries in both adults and children [37]. In Thessaly, the area of origin of the population of the present study, the overall prevalence of obesity was 26.6% whereas the prevalence of overweight was 39.4% and the mean value of BMI in that study was 27.5 kg/m^2^ [38]. Therefore, our study population is rather representative of the general population of the area of Thessaly in terms of obesity. Moreover, the fact that the significant differences of FFMI in all groups remained after the additional analysis with proper adjustments for the aforementioned confounders, further supports the need for evaluation of body composition in patients with SRA.

Another potential limitation is the use of BIA as a method for the estimation of FFM. It has been proposed that in COPD patients the method of choice for body composition assessment should be dual energy X ray absorptiometry (DEXA) [39], although in a previous study it was shown that DEXA did not differ from anthropometric measurements in COPD patients, and DEXA did not differ with either anthropometric or BIA measurements in healthy subjects [40]. In another study, Lerario et al. showed that both BIA and anthropometry presented good reliability and correlation with DEXA in COPD patients [34]. Despite the wide evaluation of those methods in COPD patients and healthy controls, there are no data in asthmatics, and further studies validating our results using DEXA are warranted.

In conclusion, in the present study we have shown that in a population of healthy smokers and patients with COPD and asthma without comorbidities, SRA is related to the presence of decreased FFMI that is comparable to that of GOLD stage IV COPD. The impact of this observation on asthma outcomes should be further investigated in large prospective studies.
